# Long-Term Thermal Aging of Modified Sylgard 184 Formulations

**DOI:** 10.3390/polym13183125

**Published:** 2021-09-16

**Authors:** Zachary Brounstein, Jianchao Zhao, Drew Geller, Nevin Gupta, Andrea Labouriau

**Affiliations:** 1 Los Alamos National Laboratory, Los Alamos, NM 87545, USA; zrbrounstein@lanl.gov (Z.B.); dgeller@lanl.gov (D.G.); nevin5@lanl.gov (N.G.); 2 Department of Nanoscience and Microsystems Engineering, University of New Mexico, Albuquerque, NM 87131, USA; 3 Department of Chemical Engineering, University of Michigan, Ann Arbor, MI 48109, USA; jczhao@umich.edu

**Keywords:** Sylgard 184, PDMS, thermal aging, gas evolution, accelerated aging, gel point

## Abstract

Primarily used as an encapsulant and soft adhesive, Sylgard 184 is an engineered, high-performance silicone polymer that has applications spanning microfluidics, microelectromechanical systems, mechanobiology, and protecting electronic and non-electronic devices and equipment. Despite its ubiquity, there are improvements to be considered, namely, decreasing its gel point at room temperature, understanding volatile gas products upon aging, and determining how material properties change over its lifespan. In this work, these aspects were investigated by incorporating well-defined compounds (the Ashby–Karstedt catalyst and tetrakis (dimethylsiloxy) silane) into Sylgard 184 to make modified formulations. As a result of these additions, the curing time at room temperature was accelerated, which allowed for Sylgard 184 to be useful within a much shorter time frame. Additionally, long-term thermal accelerated aging was performed on Sylgard 184 and its modifications in order to create predictive lifetime models for its volatile gas generation and material properties.

## 1. Introduction

Sylgard 184 is commercial high-performance silicone elastomer comprised of poly(dimethyl siloxane) (PDMS) and other silicon-based compounds originally developed by Dow Corning Corporation. Due to its optical transparency [1], thermal stability [2], mechanical advantages [3], and resistance to oxidation and hydrolysis [4], Sylgard 184 has been used as a potting material in numerous applications, spanning microelectromechanical systems (MEMS) [5,6], electronic devices, and aerospace adhesives [7]. It is fabricated by mixing a prepolymer base (Part A), also referred to as the elastomer resin, and curing agent (Part B), also referred to as the hardener [8]. What separates Sylgard 184 from PDMS is that there are additional components other than the siloxane units in parts A and B. Besides siloxane units, the prepolymer base comprises a vinylated and methylated surface-modified silica filler component and vinyl-terminated and branched siloxane components [9]. The curing agent also contains linear and cyclosiloxanes, the modified silica filler, and platinum to catalyze the hydrosilylation reaction mechanism responsible for forming the crosslinked PDMS network. The hydrosilylation reaction entails Si–H groups interacting with vinyl moieties in the presence of a catalyst [10,11,12] to form new bonds between the two chemical groups. Once both parts A and B are mixed together, the polymer undergoes crosslinking and cures in 48 h with full mechanical properties being demonstrated after a week [13]. Although the standard mixing ratio of 10:1 resin to hardener produces a material that exhibits hyperelasticity, high surface adhesion, and low Young’s modulus, these properties can be altered for a variety of applications by increasing or decreasing the mixing ratio [13,14]. In addition to altering the mixing ratio, many complementary products exist for changing viscosity and curing times [15]. Accelerating the curing time is routinely performed by curing at higher temperatures [16,17,18]; however, for many applications this approach is not feasible because the components surrounding Sylgard 184 can be heat-sensitive. One available commercial product, DOWSIL 3-6559 Cure Accelerator, which includes a platinum catalyst and oligomeric, vinyl-terminated PDMS, can accelerate room-temperature curing of silicone rubbers. Although this commercial product exists, it is desirable to tune the kinetics of the hydrosilylation reaction with controlled additions of either well-defined vinyl or silane moieties to accelerate room-temperature curing.

In addition to understanding and altering the rate at which Sylgard 184 cures, evaluating the material performance over its lifetime is necessary for its safe and predictable use. Because of its ubiquity and wide range of applications, Sylgard 184, often referred to in the literature as PDMS, has been extensively studied with regards to its material properties, including how its behavior changes when combined with fillers and other polymers to make composites; these span many cutting-edge materials such as shape memory elastomers [19,20,21,22,23,24] and wearable electronics [25,26]. Despite the plethora of research conducted on PDMS and PDMS composites, there has been much less focus on investigating how the performance of Sylgard 184 changes over time. Some work examining how aged Sylgard changes includes thermally aging chemically modified Sylgard 184 [27], UV-aging PDMS insulation [28], thermally aging resin-filled PDMS elastomers [29], humidity-aging siloxane foams [30,31], and gamma irradiation-aging silicones [32]. Natural and induced aging can lead to device or application failure and could potentially introduce new hazards. One prevalent example involves volatile products from siloxane compounds. Although it is well-known and documented within the literature and industry that hydrogen generation from silicon compounds could pose serious direct and indirect hazards [17,33,34], there have been no long-term studies quantitatively measuring hydrogen generation from PDMS. Furthermore, studies have not evaluated whether other volatiles are generated in the aging of PDMS. Instead, studies that probe volatile product generation of silicone compounds focus on induced thermal decomposition [35,36,37,38,39,40,41]. Additionally, while volatile gas evolution may pose a serious problem, of prime concern is how the material itself changes over its lifetime; yet, there is much more literature on short-term behavioral and property changes. Thus, volatile product generation, taken together with the long-term behavior of PDMS, represents a significant gap in knowledge and the literature.

The present study is an investigation aiming to solve the three problems that have been previously described. First, Sylgard 184 was modified with well-known compounds to accelerate the curing reaction at room temperature. Our team had previously worked on room-temperature curing of Sylgard 184, which resulted in finding that incorporating 0.1–0.3 wt% Ashby–Karstedt catalyst accelerated its curing time and improved its mechanical properties [13]. Additionally, we found that the incorporation of tetrakis (dimethylsiloxy) silane (TDS) (a siloxane-based silane crosslinking agent) was able to reduce the curing time significantly. Four different formulations of Sylgard 184, comprising a control and combinations of the Asbhy–Karstedt catalyst and TDS, were studied for their curing time and other initial properties. Second, thermal accelerated aging was performed on the formulations in isothermal conditions from 25 °C to 90 °C for up to twelve months. Long-term material performance was evaluated and modeled to predict behavior based on this data. Third, headspace analysis was conducted on two of the formulations to determine which volatile components were being generated during aging. Inert and oxygen-rich atmospheres were used to probe how the surrounding environment might affect the materials’ properties. Chemical, mechanical, and thermal properties were investigated to produce a thorough assessment of the lifetime performance of Sylgard 184. Thus, this study is a novel investigation into the comprehensive, long-term aging behavior of Sylgard 184 and its fast-curing formulations.

## 2. Materials and Methods

### 2.1. Materials

Dow SYLGARD™ 184 Silicone Elastomer was formulated from a base agent and curing agent, both of which were supplied by Ellsworth. Ashby–Karstedt catalyst (platinum-cyclovinylmethyl- siloxane complex; 2% Pt(0) in cyclomethylvinylsiloxanes) was supplied by Gelest. Tetrakis (dimethylsiloxy) silane (TDS) was supplied by Gelest. Ultra-high purity nitrogen was supplied by Airgas. HPLC-grade chloroform and toluene were supplied by Thermo Fisher Scientific.

### 2.2. Sylgard 184 Formulations

A ratio of 10:1 *w*/*w* was used for the base agent and curing agent, respectively, to make the standard Sylgard 184 framework. All samples were made from the same batch. It should be noted that the formulations must be made in a specific order: the TDS crosslinker and Ashby–Karstedt catalyst, when used in the formulations, are added to the elastomer base and curing agent, respectively. This is because of what they resemble in Parts A and B; TDS is chemically similar to the branched silane found in Part A while the Ashby–Karstedt catalyst is chemically similar to the cyclosiloxane and Pt found in Part B. Incorporating the additional compounds into dissimilar parts will result in the hydrosilylation reaction occurring before the prepolymer base and curing agent are added, yielding a non-uniform elastomer matrix. The TDS and Ashby–Karstedt catalyst compounds were always added in proportion to the total mass of the standard Sylgard 184 framework. For the samples made in this study, the order (if the component was present) went: (1) TDS; (2) Elastomer base; (3) Curing Agent; and (4) Ashby–Karstedt catalyst. This present study investigates the material properties of four different formulations of Sylgard 184 that are listed in Table 1. Once all the constituents were added together, the mixture was placed in a THINKY ARV-310 planetary mixer for two minutes at 2000 rpm at a vacuum of 0.2 psi. After pouring the resulting blend into the desired mold, the polymer solution was cured in a 25 °C oven for one week.

### 2.3. Thermal Accelerated Aging

Determination of long-term material performance and behavior of polymers has been investigated using the concept and methodology of thermal accelerated aging [42,43,44,45,46,47,48]. After samples had reached their gel point and fully cured, which was determined by rheology and the technical data sheet, respectively, in a 25 °C oven, they were grouped to be thermally aged. Six ovens were kept isothermal at 25 °C, 50 °C, 60 °C, 70 °C, 80 °C, and 90 °C. Samples that did not have TDS added (Control and 0pt2 formulations) were placed in sealed canisters. Each non-TDS formulation was placed in an individual canister per oven above 25 °C, which made up ten canisters. Samples were placed such that they did not have contact with the metal canisters. After they were securely sealed and fastened, the canisters had their air evacuated, creating a vacuum around 10^−3^ torr, and filled with ultra-high purity nitrogen to around 600 torr. An additional Control canister and 0pt2 canister went through the same process as described above, with the exception that these two canisters were kept in air at atmospheric pressure and placed in the 90 °C oven. Enough specimens were placed in the ovens so that they could be removed after aging for two, five, and twelve months. As an experimental note, two sample canisters for the 12-month aging Control (60 °C and 70 °C) leaked on their way to be analyzed and no mass spectrometry data could be recovered. Plots for the Control omit these two data points.

### 2.4. Chemical Characterization Techniques

Rheological measurements were taken with a TA Instruments Discover HR-2 Rheometer. For gel point determination, a 25 mm parallel plate attachment was used with a gap height of 1000 µm. With a strain rate of 4% and a frequency of 10 rad/s, the system was kept isothermal at 25 °C and the storage and loss moduli were measured as a function of time. The gel point, or working time, of the formulations was determined as the time elapsed from the beginning of the experiment until a crossover of the moduli was observed, where the loss modulus fell below the storage modulus.

Fourier transform infrared (FTIR) spectroscopy was used to determine the functional groups of the materials. FTIR was performed using a ThermoScientific Nicolet iS50 FT-IR with an average of 32 scans at a resolution of 4 cm^−1^ between 4000–525 cm^−1^. The measurements were performed in attenuated total reflection (ATR) mode with a diamond crystal reference. A background spectrum was taken before each measurement.

Contact angle measurements were used to determine how hydrophobicity changed over time. It has been shown that low molecular weight PDMS chains migrate to the surface, which quickly renders an altered hydrophilic surface into a hydrophobic one [49]. Static contact angle measurements were performed using a Drop Shape Analyzer (DSA30) from Krüss. An automated drop dispenser and deposition system was used to perform the measurements. A 2 μL drop of deionized (DI) water was dropped on the material surface and the static contact angle was then determined by the computer software. A minimum of four trials were performed on different parts of the sample surface and two samples from each formulation were tested, giving eight pairs of contact angles for each material. The reported values of static contact angle are an average and standard deviation of the measurements.

Solvent swelling and gel permeation chromatography (GPC) experiments were used to evaluate the crosslinking density and molecular weight of the non-bound extracts, respectively. Solvent swelling and GPC were performed using toluene and chloroform, respectively, which are good solvents for PDMS [6,50,51,52]. For GPC, 5.5 ± 5 g of each sample was swollen in 20 g of chloroform for 24 h at 25 °C. After swelling, the excess solvent that was not absorbed by the polymer was filtered using a 0.22 µm PTFE filter and then analyzed using an Agilent Infinity II instrument, with a Shodex (K-805L) column, Wyatt Technology detectors, DAWN multi-angle light scattering (MALS, 25 °C), and Optilab refractive index (RI, 30 °C). The instrument used 75 µL of each sample per experiment and ran the material through the column at a rate of 1.0 mL/min. Data was analyzed using Astra software. Values for the number average molar mass, M_n_, weight average molar mass, M_w_, and polydispersity, Đ, were obtained using the Debye model for light scattering, and a refractive index increment dn/dc = −0.0646. For solvent swelling, Sylgard 184 and its modifications were swollen to equilibrium in toluene for 24 h at 25 °C [6]. Additional experiments were run to ensure swelling equilibrium would be attained. Control samples were swollen in toluene for 1, 2, 3, 6, and 7 days and subsequently vacuum-dried. All the samples had the same relative swollen mass and relative dried mass, thus confirming that swelling equilibrium is reached after one day. After being swollen, the samples were gently patted dry to remove residual toluene before recording the swollen mass. The samples were then dried at 25 ℃ for 96 h under vacuum. 

Percent swelling from the solvent is reported as the difference between the fully swollen mass and initial mass. Percent loss from the solvent is reported as the difference between the initial mass and the vacuum dried mass. The network swelling is reported as the difference between the fully swollen mass and vacuum dried mass. Each sample was run in triplicate and standard deviation is reported as the error. Sample dimensions were 11.1 mm in diameter and 0.98 ± 0.1 mm in thickness.

The polymer volume fraction $\varphi_{p}$ was determined using the respective partial volumes of the polymer $V_{p}$ and solvent$\;V_{s}$. Because these values are not easily measured, the densities of polymer$\;\rho_{p}$ and solvent$\;\rho_{s}$ were used along with the network swelling, *S*, to obtain the volume fraction, which is shown as Equation (1). This was used to evaluate the Flory–Huggins polymer–solvent interaction parameter χ between PDMS and toluene, where many equations have been developed to describe the interaction parameter from theory and experimental methods [52,53,54,55,56,57]. Searching through the literature regarding swelling PDMS with toluene, the equation that best describes the systems in this study is a finite Taylor series as a function of the polymer volume fraction, with values shown in Equation (2) [52].

(1) $$\varphi_{p}=\frac{V_{p}}{V_{p}+V_{s}}={\left(1+S\frac{\rho_{p}}{\rho_{s}}\right)}^{-1}$$

(2) $$\chi =\chi_{0}+\chi_{1}\varphi_{p}+\chi_{2}\varphi_{p}{}^{2}=0.459+0.134\varphi_{p}+0.590\varphi_{p}{}^{2}$$

Using the interaction parameter along with the polymer volume fraction and molar volume of the solvent$\;v_{m,s}$ the average molecular weight of the polymer between crosslinks$\;M_{c}$ can be evaluated using a modified version of the Flory–Rehner equation. The standard Flory–Rehner equation is derived from thermodynamic interactions and treats the swollen polymer as a perfect network, where the numerator and denominator correspond to the elastic and mixing contributions, respectively [52,58,59,60,61]. Groups working with both theoretical and experimental methods regarding polymer swelling behavior have shown that a phantom network models a swollen polymer with better precision and as such is used in this study as Equation (3) [52,58]. The same Flory–Rehner equation can also relate the effective number of chains in the network$\;\nu_{e}$ to the average molecular weight of the polymer between crosslinks and Avogadro’s number$\;N_{A}$, shown as Equation (4). Rearranging the terms in Equation (4) produces the specific crosslink density$\;p_{x}$ of the polymer, shown as Equation (5), which describes the moles of crosslinks per mass of polymer.

(3) Mc=−12vm,sρpφp13ln(1−φp)+φp+χφp2

(4) $$\nu_{e}=\frac{\rho_{p}N_{A}}{M_{c}}$$

(5) $$\frac{\nu_{e}}{\rho_{p}N_{A}}=\frac{1}{M_{c}}=p_{x}$$

Mass spectrometry was performed on the headspace of each sealed canister using a Finnigan MAT 271 magnetic-sector mass spectrometer. In addition to the standard Faraday-cup detector, this closed-source, electron-ionization (EI) gas mass spectrometer is equipped with a secondary electron multiplier for detection of trace species. The two detectors were calibrated against standards for several permanent gases. For other gases and vapors, estimates were made from sensitivities based on the electric dipole polarizabilities of molecules, relative to those of the calibration gases [62]. Species were identified by comparison of the measured spectra against the National Institute of Standards and Technology (NIST) electron ionization catalog and the EI fragmentation patterns were used to subtract the contributions of ions of higher-mass molecules from lower-mass peaks. Raw data was in the form of parts per million (ppm) and was converted to moles using the ideal gas law and the measured pressure of the canisters.

### 2.5. Mechanical Characterization Techniques

Shore A Hardness (ShA) testing was performed on a benchtop HPE II Zwick Roell Shore A hardness tester using discs with a diameter of 29 mm and a thickness between 10 and 13 mm, because samples with a thickness greater than 6 mm reduce the error associated with the test [13]. Values were taken at equilibrium when the hardness reading had stopped changing. Hardness values were averaged using three samples that were each tested in at least three different locations. Standard deviation is reported as the error.

Tensile strength was measured using an ADMET eXpert 7601 testing system. All samples were cut into dumbbell shapes, referred to as dogbones, using a type-A specimen die following ASTM D412 standards. The samples were loaded into the uniaxial grips and then pulled to reach a break point at the speed of 3.84 mm/sec. Three trials were performed for each sample and the engineering stress, strain, and Young’s modulus are reported as averages and corresponding standard deviations.

### 2.6. Thermal Characterization Techniques

Thermogravimetric analysis (TGA) experiments were conducted to evaluate the thermal stability of the samples. TGA was performed using a TA Instrument TGA 550, Discovery Series. Samples weighing 10 mg ± 1 mg were heated at a temperature ramping of 5 °C/min to 750 °C under nitrogen passing through the furnace at 40 mL/min. The onset of thermal degradation *T_d5%_* is a measure of thermal stability and applicability, which is determined from the temperature at which the residual mass is 95% of the total. The temperature of thermal decomposition is that temperature at which the derivative graph (DTGA) is a maximum. Differential Scanning Calorimetry (DSC) experiments were conducted to determine the glass transition temperature *T_g_*, along with any phase transitions. DSC experiments were performed using a TA Instrument, DSC Q20a, Q Series. The samples, weighing between 5 and 10 mg, were cooled down at 10 °C/min to −150 °C, ramped at 5 °C/min to −100 °C, and then ramped at 10 °C/min to 30 °C. The reported glass transition temperatures are those found along the heating curve. Coefficient of linear thermal expansion (CTE) experiments were conducted using the dilatometer TA Instrument DIL 802. Cylindrical samples measuring approximately 19.00 mm in length and 4.5 mm in diameter were placed next to a fused silica standard and heated under nitrogen at a rate of 5 °C/min until the furnace reached 150 °C. The CTE value *α*, evaluated using Equation (6), was provided by the instrument’s software based on the change in length Δ*L*, change in temperature Δ*T*, and original length *L*_0_. Average values and standard deviations are reported from CTE values after they reached a stable number, typically after the sample reached above 40 °C.

(6) $$\alpha =\frac{1}{L_{0}}\frac{\Delta L}{\Delta T}$$

## 3. Results and Discussion

### 3.1. Material Properties of Pristine Samples

**Figure 1:**
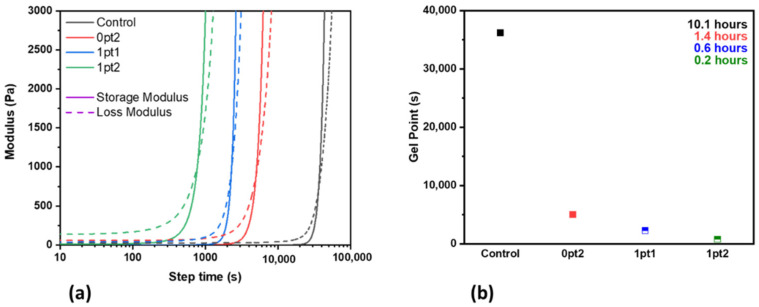
Rheology of the four formulations: (**a**) Storage and loss moduli, and (**b**) Gel point.

Rheology performed on the four formulations (Control, 0pt2, 1pt1, and 1pt2) shows that the gel point, which is when the polymer forms a 3D network and can be used as a comparison for curing time, decreased significantly from the Control to any of the formulations with the Ashby–Karstedt catalyst. The reduction in the time for the gel point to appear was even more significant with samples containing TDS. The rheology results, shown in Figure 1, demonstrate that a small addition to the formulation can speed up the curing of the thermoset elastomer. Compared to the unmodified Control, the 0pt2, 1pt1, and 1pt2 formulations had a reduction in gel point time by 86%, 94%, and 98%, respectively. From this, it is inferred that a greater amount of Pt and vinyl groups within the Ashby–Karstedt catalyst contribute towards the crosslinking of the prepolymer and resin. This has also been seen when the standard 10:1 ratio of base to hardener is lowered so that there is more platinum that can react with the prepolymer, which leads to a faster curing time [63,64]. Studies that demonstrate this fact also demonstrate that Sylgard 184 prepared with the lowered ratio comes with varying mechanical properties. Additionally, from the rheology, it can be inferred that the TDS acts as a bridge between separately formed PDMS chains that would have otherwise have either not joined the network or taken much longer to do so. Thus, incorporating both the Ashby–Karstedt catalyst and TDS allows for more reaction sites and vinyl groups to participate in the crosslinking reaction and make a more connected network.

**Figure A1:**
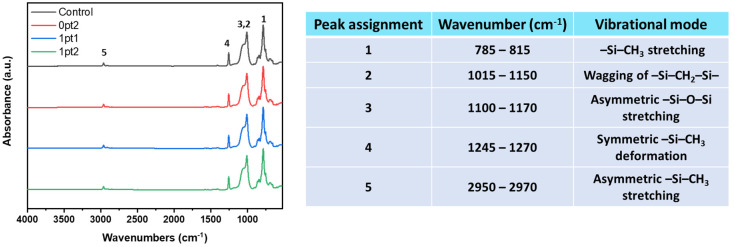
FTIR spectra of formulations along with their peak assignments.

**Figure A2:**
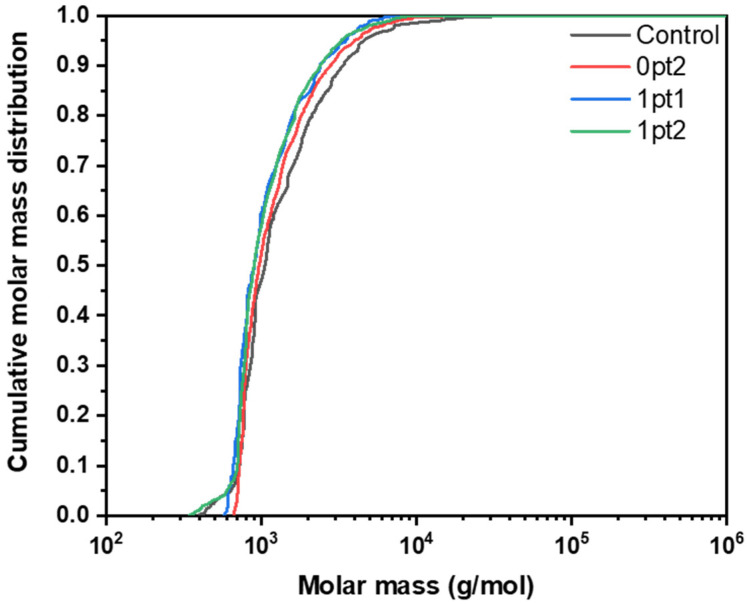
Cumulative molar mass distribution of the extracts from pristine samples.

The FTIR—ATR spectra for Sylgard 184 is shown in Appendix A as Figure A1 along with five IR peak assignments. Comparing the FTIR spectra for all four formulations reveals distinct regions in similar locations with similar intensities. Thus, the modified formulations do not appear to differ from the Control with respect to chemical functional groups. GPC experiments, which evaluated the molecular weights and distributions of the extractable material in the formulations, showed three peaks for the samples that occurred around two, four, and ten minutes, which correspond to weights of 10^7^, 10^6^, and 10^3^ g/mol, respectively. All the formulations exhibited these peaks, and the cumulative molar mass distributions for the samples, shown in Appendix A as Figure A2, exhibited similar results for each formulation.

With regards to surface characteristics, Sylgard 184 is naturally hydrophobic due to the methyl end groups on the polymer chain, however it can easily be chemically and physically altered to exhibit other surface characteristics [65]. The Control samples demonstrated a static contact angle with water of 110° ± 9°, which agrees with the literature values reporting measurements of 109°. Measured static contact angles for the modifications are 110° ± 11° for 0pt2, 110° ± 4° for 1pt1, and 114° ± 13° for 1pt2, thus showing that hydrophobicity is invariant across the formulations.

**Figure 2:**
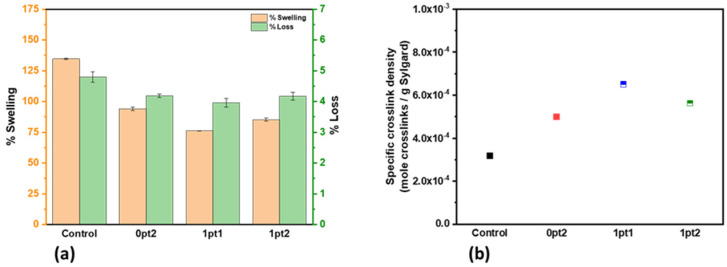
Solvent swelling experiments of pristine samples in toluene: (**a**) Swelling and loss percent for the formulations and (**b**) Specific crosslinking density$\;p_{x}$ for the formulations.

Swelling experiments on the pristine specimen showed that while all the samples absorbed enough toluene to nearly double their mass, the Control formulation clearly absorbed the most solvent. Indeed, the Control formulation absorbed enough solvent to increase its mass by over 125%, while the modified samples were under 100%. Mass losses after drying were all similar across the samples, measuring at around 4%. Comparisons of swelling between samples are shown in Figure 2a. The measured density of the samples averaged to 0.98 g/cm^3^ and is used throughout this study. While the manufacturer reports a density of 1.03 g/cm^3^, others such as Olima have reported 1.18 g/cm^3^ [3]. Equation (5) provides the specific crosslink densities of the pristine samples, which are shown in Figure 2b. A decrease in swelling corresponds to an increase in crosslink density; thus, Figure 2a,b both describe a similar phenomenon, where the modified samples have a greater specific crosslink density than the Control.

**Figure 3:**
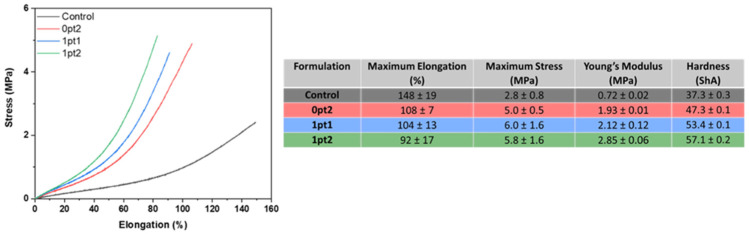
Mechanical properties of pristine samples.

The mechanical data shows the first recognizable distinction between the four formulations; the modified samples became harder and less flexible on a qualitative basis compared to the Control. Tension tests performed on the dogbone samples confirmed this quantitatively. The results of the tension and hardness tests, shown in Figure 3, demonstrate that all the modified samples become stiffer and less flexible by measure of maximum elongation and stress, and Young’s modulus. Hardness measurements, by way of Shore A Hardness durometry, also quantitatively prove that the modified pristine samples are more resistant to indentation than the Control. These results correlate with Figure 2, where an increase in the specific crosslink density is associated with an increase in hardness and Young’s modulus.

**Figure 4:**
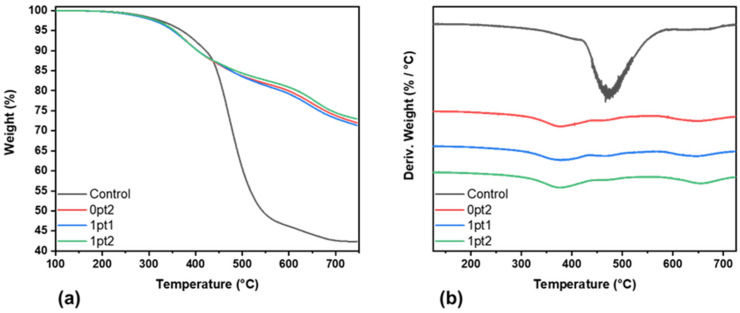
Thermal stability of pristine samples: (**a**) TGA curves and (**b**) DTGA curves magnified.

All four formulations exhibit thermal degradation at similar temperatures, as observed in the TGA and DTGA curves in Figure 4. Figure 4b shows all the DTGA curves, magnified four times so that the peaks are more readily observed. Differences in thermal stability between the modified and unmodified Sylgard 184 samples were compared using the onset of thermal degradation *T_d5%_*, which is the temperature at which the materials lose 5% of their initial mass. While the Control sample exhibited an onset degradation temperature of 370 °C, the *T_d5%_* for all the modified samples were similar, with values of 355 °C for 0pt2, 352 °C for 1pt1, and 355 °C for 1pt2—a 15 °C decrease. Additionally, the modified formulations had similar thermal degradation mechanisms, where residual masses were comparable (Figure 4a) and thermal decomposition peaks were located at the same temperatures (Figure 4b), while the Control sample sharply deviated after the first thermal degradation mechanism. Furthermore, the Control sample had a 30 wt% lower residual mass than the modified formulations, indicating that more volatile products were formed due to thermal decomposition. The high residual mass exhibited in Figure 4a has also been observed in the literature for Sylgard 184 and some silicone polymers [3,66,67,68]. The greatest difference in the degradation profiles was in the quality of the peaks. In the DTGA curves for the modified samples, three peaks are observed, with the greatest relative peak being the first one, which occurs around 360 °C and is comparable to literature values for PDMS decomposition [36,37,69]. Additionally, all three peaks occur in similar temperature regions for the modified samples. This is in contrast to the Control, where the second peak is so pronounced that it obscures the others in the pristine sample. The decomposition peaks from the DTGA curves correspond to approximately 360 °C, 470 °C, and 650 °C in nitrogen. These have been observed before and have been attributed to the depolymerization process of the chain backbone [3,41,70]. Figure 4 demonstrates that the pristine Control samples are initially more thermally stable, and the chain backbone can withstand greater thermal excitations than the modified samples; however, once the depolymerization process begins to occur, the Control samples rapidly decompose and form volatile products, while the modified formulations decompose less readily.

CTE values were 266 ppm K^−1^ for the Control, 250 ppm K^−1^ for 0pt2, 203 ppm K^−1^ for 1pt1, and 207 ppm K^−1^ for 1pt2. Along with the original manufacturer of Sylgard 184 claiming that it has a coefficient of linear thermal expansion of 340 ppm K^−1^ [8], Liu et al. reports 300 ppm K^−1^ [71], Müller et al. reports 310 ppm K^−1^ [72], and Kong et al. reports 362 ppm K^−1^ [73]. The measured CTE values show an improved performance with the modified samples compared to the Control for applications using Sylgard 184, such as micromechanical devices and optical instruments. This is because the polymer is frequently in contact with metals or materials that conduct heat and exhibit low thermal expansion coefficients. Equipment and tools such as these frequently fail due to thermal expansion mismatch, which is why methods that reduce the thermal expansion coefficient are thought to improve performance. The range of CTE values measured for the four formulations and found from the literature demonstrates that while the CTE of Sylgard 184 is significantly higher than most polymers, metals, and other materials, there is a large variability in this property, likely due to fluctuations in different batches.

Glass transition temperatures for all the formulations were similarly at −120 °C. No crystallization or melting peaks were observed in any heating or cooling cycles, even when the range was extended from −150 °C to 200 °C. This agrees with previous results where Sylgard 184 has a *T_g_* around −125 °C and exhibits no crystallization peaks, making the material a fully amorphous polymer [3].

### 3.2. Mechanisms for Aging under Nitrogen

**Figure 5:**
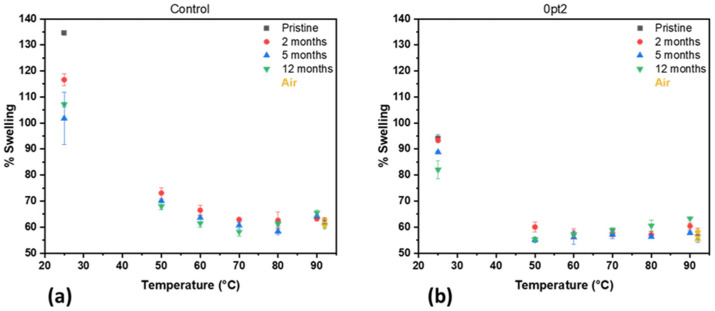
Swelling of thermally aged samples: (**a**) Control and (**b**) 0pt2.

The Control and 0pt2 samples underwent thermal accelerated aging under a nitrogen atmosphere. Swelling data for the aged Control and 0pt2 samples are shown in Figure 5a,b, respectively. An immediate observation from Figure 5 is that both formulations undergo a post-curing reaction, where the swelling at room and elevated temperatures indicates that the crosslink density increases. A second observation is that after two months of thermal accelerated aging, the swelling of both the Control and 0pt2 samples reaches a plateau, revealing that the majority of the hydrosilylation reaction has run to completion. The third observation, which highlights the difference between the Control and 0pt2 samples, is that the 0pt2 samples experience a lower overall change in swelling than the Control, which occurs more rapidly. A final observation is from the samples aged in air at 90 °C, which are colored yellow and offset in Figure 5 at 92 °C, solely for legibility. Notice that the samples aged in air at 90 °C exhibit similar swelling behavior to those samples aged in nitrogen at 90 °C.

The hydrosilylation reaction responsible for the curing of Sylgard 184 creates crosslinks but does not generate volatile products. Analysis of the headspace of the sealed canisters via mass spectrometry allows for an evaluation of volatile products and can assist in proposing mechanisms responsible for the reactions. The most common evolved products were hydrogen, methane, and ethane, with the former two making up the vast majority of volatiles detected. These can be produced from a proposed reaction mechanism involving water interacting with Si–H groups; although the hydrosilylation reaction is an addition reaction between Si–H and vinyl groups in the presence of a catalyst, notably Pt, water can also attack Si–H to form Si-OH in the presence of the same catalyst [17,34]. Additionally, siloxanols can be created from the hydrolysis of PDMS, where a water molecule attacks a siloxane chain, resulting in a chain scission and two hydroxyl-terminated chains [74,75,76]. If a siloxanol encounters a silane, a new crosslink will form between the two moieties and H_2_ gas will be produced. If, however, a siloxanol encounters a Si–CH_3_ group instead, a new crosslink will form between the two moieties and CH_4_ gas will be produced [77,78]. Ethane is produced in a similar manner, except with a Si–C_2_H_5_ group encountering a siloxanol.

**Figure 6:**
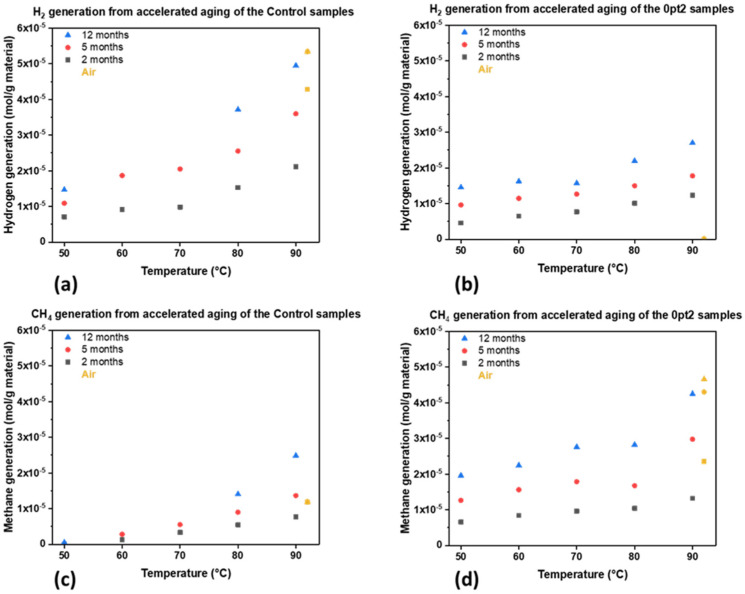
Measuring the volatile products that evolved while undergoing thermal accelerated aging on a per-mass-of-Sylgard basis: (**a**) hydrogen generation from the Control; (**b**) hydrogen generation from 0pt2; (**c**) methane generation from the Control; and (**d**) methane generation from 0pt2.

The cumulative amounts of hydrogen and methane in the Control and 0pt2 samples are shown in Figure 6. Furthermore, samples that were aged at 90 °C in air are included as before: yellow and offset in Figure 6 to 92 °C, solely for legibility. Two initial observations stand out, which are that as time progresses and temperature increases, the cumulative amount of volatiles becomes greater. Additional interesting observations can be made when comparing volatile product generation between the two formulations. One would be that there is more hydrogen generation for the Control than 0pt2 samples. The other would be the case for methane generation, where 0pt2 generated much more methane than Control samples.

**Figure A3:**
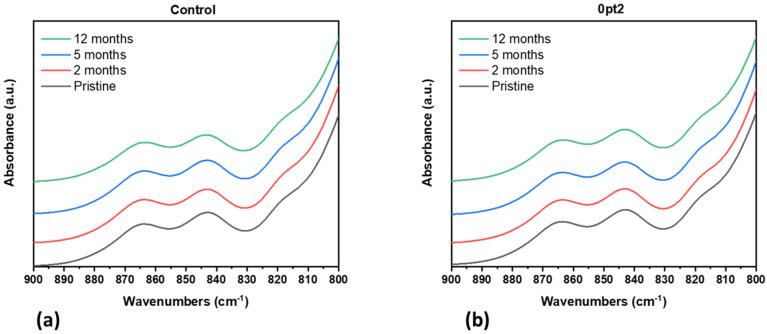
FTIR absorbance spectra of pristine and 50 °C aged samples showing peaks at 845 cm^−1^ and 865 cm^−1^, which correspond to siloxanol groups (Si–OH): (**a**) Controls and (**b**) 0pt2.

The proposed mechanism, which depends on trace water, should be able to be indirectly observed via Si–OH bonds. Based on FTIR data, there are peaks around 845 cm^−1^ and 865 cm^−1^ (shown in the Appendix A as Figure A3), which correspond to Si–O stretching in Si–OH [65]. Additionally, mass spectrometry revealed small amounts of trace water (in ppm levels) in the headspace. Although the FTIR data was not quantitatively analyzed, reviewing the spectra and mass spectrometry of the samples lends credence towards this proposed mechanism. The addition of the 2% Pt Ashby–Karstedt catalyst, which introduces more vinyl groups and thus promotes crosslinking, leaves fewer available Si–H groups for H_2_ generation. Thus, the 0pt2 samples should be expected to generate less H_2_ gas than the Control samples but generate more CH_4_. Additionally, observe how the volatile gas production in Figure 6 increases with temperature; greater thermal energy leads to more chain mobility and thus more interactions between siloxanes, silanes, and siloxanols. Thus, the proposed mechanism provides an explanation for the observed data.

It is possible to draw conclusions about the specific crosslink density using Equation (5). Based on the proposed mechanisms, all the crosslinks in the polymer network are assumed to be from the hydrosilylation (cure and post-cure) and trace water-promoted reactions. Treating the specific crosslink density as separable with regards to reactions that generate crosslinks, it can be thought of as the sum of the specific crosslink density due to hydrosilylation$\;p_{x}^{hydrosilylation}$ and water reactions$\;p_{x}^{radical/water}$ as shown in Equation (7). Because the hydrosilylation reaction does not generate volatile gas, each molecule of volatile gas is produced as a result of a crosslink formed from a trace water reaction. Thus, summing the moles of hydrogen, methane, and ethane measured via mass spectrometry yields the amount of crosslinks formed due to trace water. Additionally, Equation (7) can be used to define the fraction of specific crosslink density due to trace water$\;\eta_{water}$ as shown in Equation (8).

(7) $$p_{x}=p_{x}^{hydrosilylation}+p_{x}^{water}$$

(8) $$\eta_{water}=\frac{p_{x}^{water}}{p_{x}^{hydrosilylation}+p_{x}^{water}}$$

**Figure 7:**
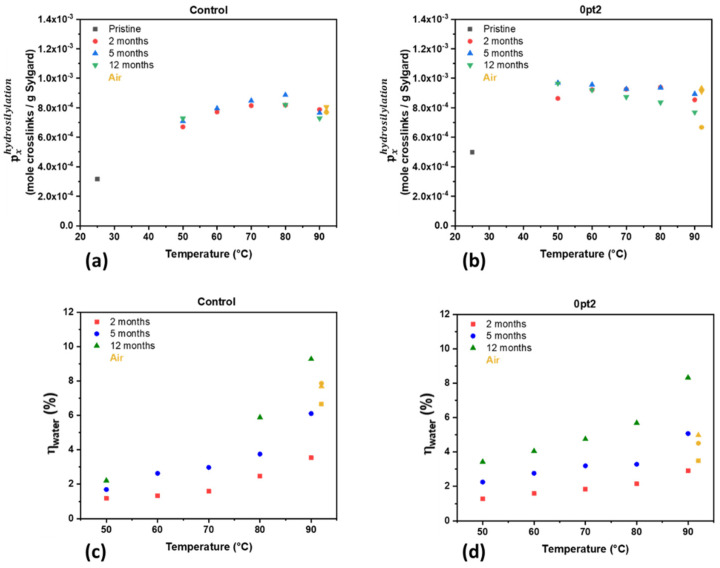
Specific crosslink density: (**a**) Crosslinks due to hydrosilylation for Controls; (**b**) Crosslinks due to hydrosilylation for 0pt2; (**c**) Water fraction for Controls; and (**d**) Water fraction for 0pt2.

**Figure A4:**
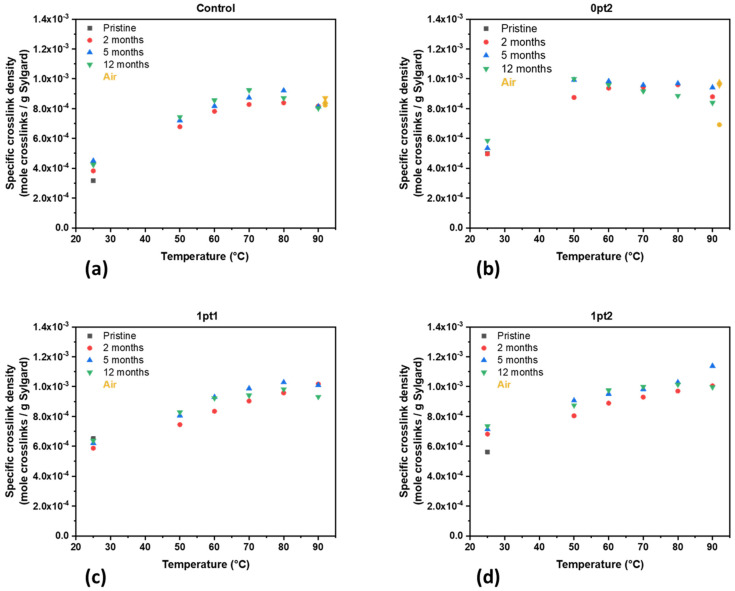
Specific crosslink densities of thermally aged samples: (**a**) Controls; (**b**) 0pt2; (**c**) 1pt1; and (**d**) 1pt2.

Equation (8) can now be used to observe how changes in the specific crosslink density for the aged formulations (shown in the Appendix A as Figure A4) are due to various reactions. Using the mass spectrometry data along with the swelling data allows for an understanding of crosslinking behavior due to the cure, post-cure, and trace water reactions, which is shown in Figure 7. The specific crosslink densities due to hydrosilylation reactions for Controls and 0pt2 (Figure 7a,b) show that the amount of crosslinks per mass of Sylgard does not change significantly—which mimics the swelling data. Indeed, both the Control and 0pt2 samples seem to level off between 0.7–0.8 mmol of crosslinks due to curing and post-curing per gram of Sylgard. Additionally, 0pt2 samples converge to the plateau at a much earlier time than the Control, whereas after two months the 0pt2 samples do not exhibit any significant change in specific crosslink density. Furthermore, the fraction of specific crosslink density due to trace water reactions (Figure 7c,d) increases over time and temperature. Thus, while the post-curing reaction ceases after two months and stops contributing to the specific crosslink density, the trace water reactions continue to promote crosslinking and gas evolution.

**Figure A5:**
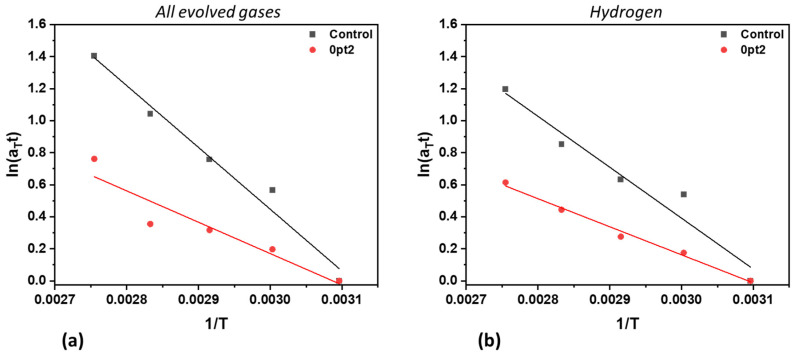
Validity of the TTS model for mass spectrometry data, where $\ln\left(a_{T}t\right)$ and  1T display a linear relationship for (**a**) all evolved gases and (**b**) hydrogen.

Traditionally, time–temperature superposition (TTS) is performed in relation to stress, creep, or dynamic loading, but in essence, material properties need to be measured and compared to a reference for the principle to be applicable. Because the aging temperatures used in this study are well below that of any thermal decomposition and well above any thermal phase transition, mass spectrometry can be used according to TTS and a master curve can be created as a function of shift time. The lowest temperature used for mass spectrometry, 50 °C, was the reference temperature$\;T_{o}$. After two months, the shift factor$\;a_{T}$ was defined in terms of the moles of gas produced 𝓃, shown in Equation (9), which allowed for a new axis of shift time$\;a_{T}t$ to be used, where all the data can be superimposed. For specific degradation or reaction mechanisms, if Arrhenius behavior was followed, the apparent activation energy *E* can be defined in terms of the universal gas constant *R*, the shift factor, and differences in reciprocal temperatures in Kelvin, as shown in Equation (10). Performing TTS with mass spectrometry data can be validated with these equations if there is a linear relationship between$\;\ln\left(a_{T}t\right)$ and 1T, which is shown as Figure A5.

(9) $$a_{T}=\frac{{𝓃}_{species}\left(T\right)}{{𝓃}_{species}\left(T_{0}\right)}$$

(10) $$E=\frac{R\ln\left(a_{T}\right)}{\frac{1}{T_{o}}-\frac{1}{T}}$$

**Figure 8:**
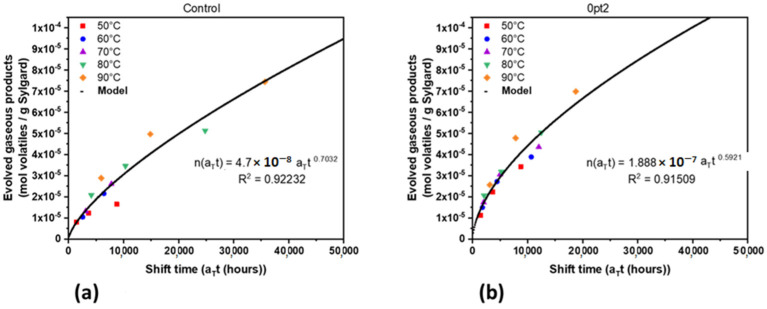
Time–temperature superposition master curve models of the three major evolved gaseous products: (**a**) Controls and (**b**) 0pt2.

By plotting all the evolved gases (hydrogen, methane, and ethane) for the Control and 0pt2 samples against shift time, a trend can be observed. Using statistical regression, the superposition data was modeled with a power function. These are shown in Figure 8 along with the coefficient of determination$\;R^{2}$. Although Figure 6 shows that the Control samples generate more hydrogen than 0pt2, enough methane and ethane were generated by 0pt2 to have its model appear greater for overall volatile gas production. Power functions were used in the superposition model because there were few volatiles evolved at the beginning of aging but gradually increased as more time went on. The average activation energy for the Control and 0pt2 samples was 38.2 kJ/mol and 15.5 kJ/mol, respectively, which is not surprising given that the shifted time values are much closer together for 0pt2 than the Control.

**Figure A6:**
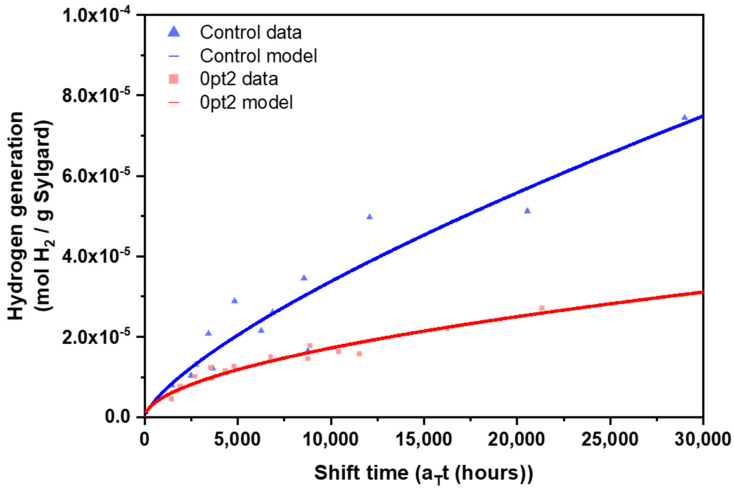
Hydrogen generation for the Control and 0pt2 formulations. Included is the shifted data and the model. For the Control, the equation is $𝓃\left(a_{T}t\right)=4.22\times 10^{-8}a_{T}t^{0.7257}$ and for the 0p2, the equation is $𝓃\left(a_{T}t\right)=1.20\times 10^{-7}a_{T}t^{0.5391}$.

Because hydrogen generation is a major known cause of hazards when dealing with siloxanes and silicon-based polymers in general, models were also developed for the Control and 0pt2 samples with regards to hydrogen evolution. Using the same procedure as detailed above for all major volatiles, the power law models were derived. For the Controls, the coefficient and exponent in shift time are 4.22 × 10^−8^ and 0.7257, respectively. For the 0pt2, the coefficient and exponent in shift time are 1.2 × 10^−7^ and 0.5391, respectively. Plots of these models are shown together in the Appendix A as Figure A6. The average apparent activation energy for the Control and 0pt2 samples was 33.4 kJ/mol and 14.4 kJ/mol, respectively.

It is apparent from Figure 6 that the Control samples generated more hydrogen than 0pt2 samples, while 0pt2 samples generated more methane than Control samples. Interestingly, an oxygen-rich environment (air) causes more hydrogen to be produced by the Controls than in nitrogen, while almost no hydrogen is present for two, five, and twelve months of thermal accelerated aging of the 0pt2 samples. Additionally, an oxygen-rich environment causes more methane generation in the 0pt2 samples than an inert environment, while methane ceases to generate in an oxygen-rich environment after two months of aging for the Controls. Indeed, an initial amount of methane was produced after two months at 90 °C in air, but afterwards the cumulative amount barely increases.

Therefore, the presence of oxygen also has an influence on volatile products. It appears that whichever gas species is more prominent (hydrogen or methane), oxygen increases its production while it sequesters the other evolved gas (methane or hydrogen). These observations can be seen when comparing Figure 6a,d and Figure 6b,c. These novel data and results suggest that a preference can be chosen for both the formulation of Sylgard 184 and the atmosphere it is stored in, to selectively generate or suppress certain evolved permanent gases.

### 3.3. Material Properties of Aged Samples

**Figure 9:**
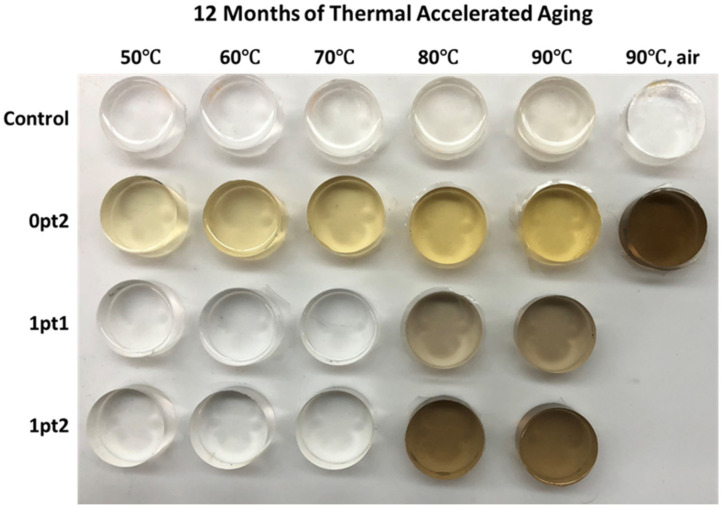
Discoloration of the four formulations after 12 months of thermal accelerated aging.

Figure 9 shows the four formulations after twelve months of thermal accelerated aging. There is a general trend, where aging at higher temperatures leads to a greater amount of discoloration. This is exhibited in every formulation and throughout the interior of the samples, although the extent of discoloration is more pronounced in the modified specimen. The clear-to-yellow discoloration phenomenon of silicones is known to be caused by platinum–complex interactions and does not affect material performance [79,80,81,82]. Interestingly, all samples produce an obvious yellowing at 80 °C if they haven’t already been discolored. The greater discoloration of the modified samples can be explained due to the addition of the Asbhy–Karstedt catalyst, which introduces more platinum to complex. Furthermore, the addition of TDS prevents discoloration up to 70 °C when comparing 1pt1 and 1pt2 samples to 0pt2. The Control samples, which have no additional platinum added, exhibit the least discoloration. Comparing aging in nitrogen versus air, while the 0pt2 samples were all yellow, the samples aged in air were the deepest brown color, and the Control sample aged in air did not experience a yellowing effect at all.

From a chemical structure perspective, the FTIR data show that the peaks for all formulations make a near perfect superposition when comparing aging time and temperature. Additionally, no new peaks have formed. Thus, any chemical changes within the material are the product of the rearrangement of bonds, due to furthering the hydrosilylation reaction and other crosslinks due to trace water. Over time, all the aged formulations still exhibited contact angles above 90°, thus they continued to demonstrate hydrophobicity. No significant changes to hydrophobicity were observed during the aging process.

The 1pt1 and 1pt2 formulations exhibited a decrease in solvent swelling similar to the others shown in Figure 5, where there was an increase in the specific crosslink density for all the formulations over their aging conditions. It should be noted that during swelling experiments, the mass of the extracts for all the samples changed little from the pristine, exhibiting values around 3.5%. For swelling, there is significantly less change after two months of aging, where considerable overlap occurs between five and twelve months. The final swelling values, taken from samples aged at 90 °C for 12 months in air, were 60%, 56%, 58%, and 56% for the Control, 0pt2, 1pt1, and 1pt2 samples, respectively. For the same samples, the final specific crosslink densities were 8.7 × 10^−4^, 9.6 × 10^−4^, 9.3 × 10^−4^, and 1.0 × 10^−3^ mole crosslinks/gram Sylgard for the Control, 0pt2, 1pt1, and 1pt2 samples, respectively. Evaluating the relative changes for swelling and specific crosslink density demonstrated that the Control formulation experienced a greater overall change compared to the modified samples for both quantities.

Similar trends were observed for mechanical properties. Like the swelling data, there were no statistically significant differences between the samples aged at 90 °C in nitrogen or air; the atmosphere did not affect the final values of the mechanical properties. Obtaining values from the samples aged at 90 °C for 12 months in air, the final mechanical properties are displayed in Table 2. Similar to swelling behavior, the Control samples exhibited the greatest percent change overall, which can be seen when comparing the values from Table 2 with Figure 3. In general, all the formulations became harder and stiffer over time, along with the materials not being able to be stretched as much as they originally could without breaking. As an example, the Control, 0pt2, 1pt1, and 1pt2 samples increased in Shore A Hardness after thermal aging by 72%, 40%, 26%, and 20%, respectively. Thus, final values for maximum elongation decreased, while Shore A Hardness, maximum stress, and Young’s modulus increased.

**Figure 10:**
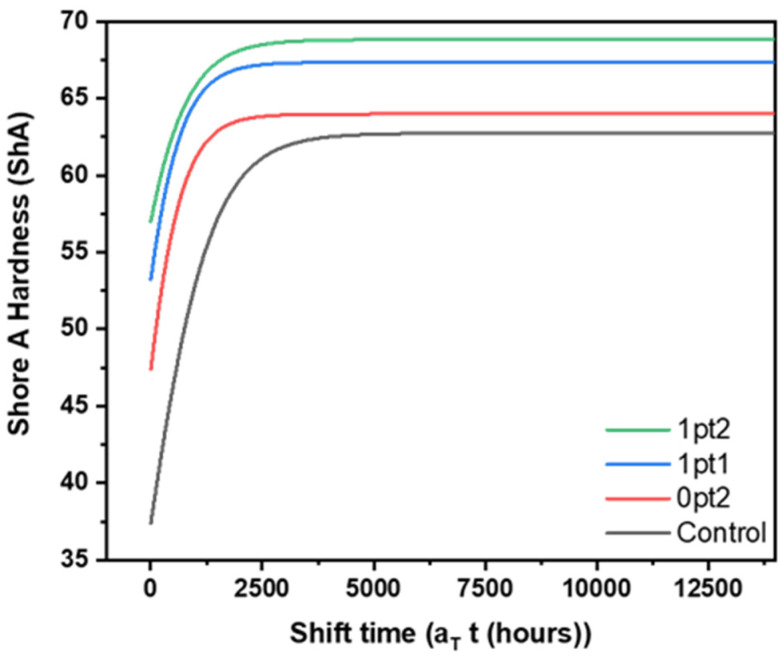
Time–temperature superposition master curve models of Shore A Hardness for the four thermal accelerated aged Sylgard 184 formulations.

**Figure A7:**
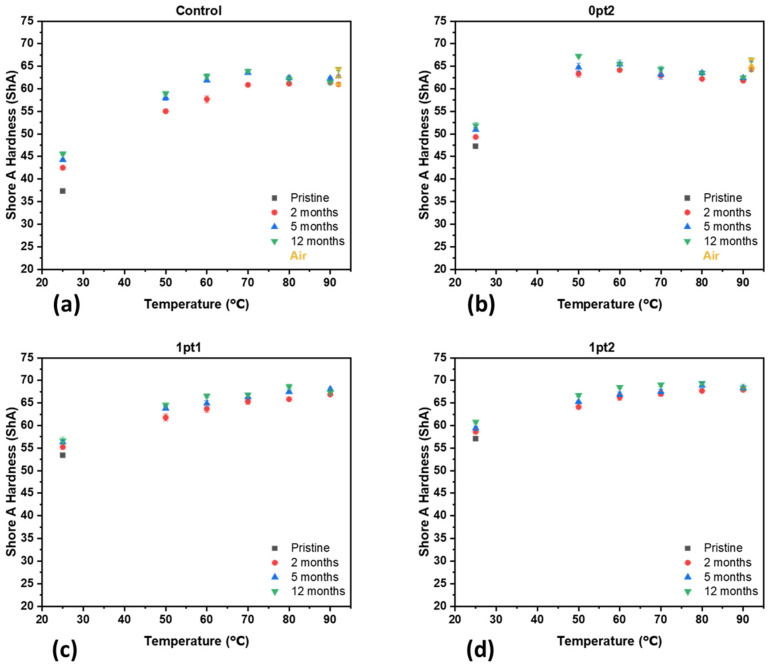
Shore A Hardness of thermally aged samples: (**a**) Controls; (**b**) 0pt2; (**c**) 1pt1; and (**d**) 1pt2.

**Figure A8:**
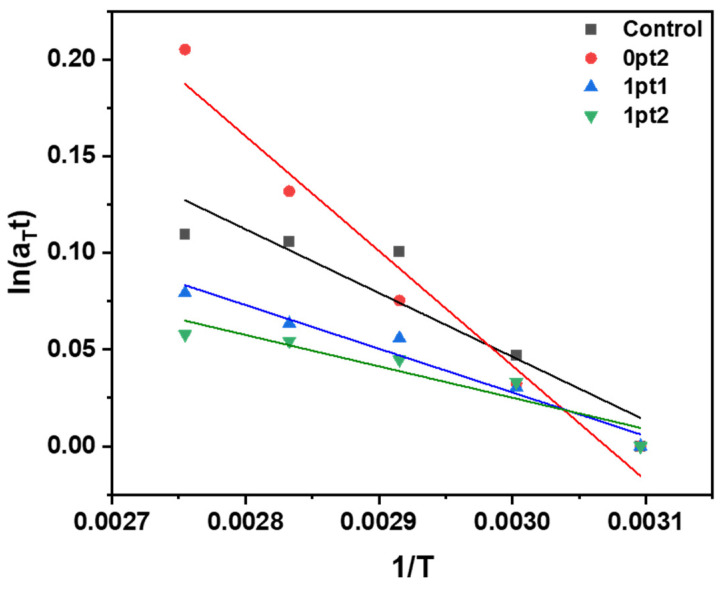
Validity of the TTS model for Shore A Hardness data, where $\ln\left(a_{T}t\right)$ and 1T display a linear relationship for all modifications.

Shore A Hardness values were used for making TTS predictive master curves, which are shown in Figure 10. The reference temperature used in the procedure was 50 °C. Because hardness is generally monotonic and reaches a limit (Figure A7 in the Appendix A), a logistic curve was used to fit the shift time data using Equation (A1). The parameters of each logistic curve are given in Appendix A Table A1. An interesting observation from performing this procedure is that the apparent activation energy at a given temperature is about 20% more than the activation energy for samples aged at 10 °C higher. For the highest temperature, 90 °C, the apparent activation energies were 9.0 kJ/mol, 5.0 kJ/mol, 1.9 kJ/mol, and 1.4 kJ/mol for the Control, 0pt2, 1pt1, and 1pt2 formulations, respectively. Similar to the mass spectrometry data, a plot to validate using TTS models for hardness data is shown in Figure A8. From the TTS master curve model, aging at an elevated temperature of 50 °C would take 82 days, 39 days, 38 days, and 35 days for the Control, 0pt2, 1pt1, and 1pt2 samples, respectively, to reach 95% of their final hardness values.

**Figure 11:**
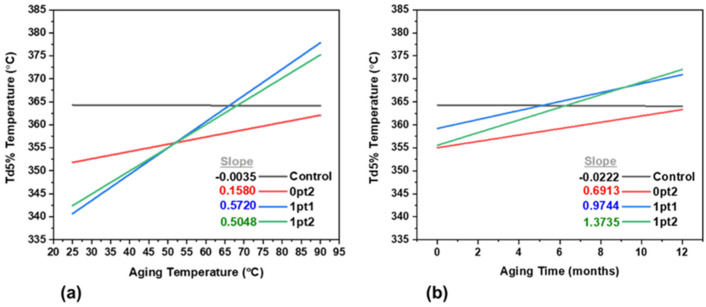
Linear regression of thermally aged samples (**a**) as a function of aging temperature and (**b**) as a function of aging time.

The onset of thermal degradation, Td5%, for the formulations changed throughout the aging process. To better visualize trends due to accelerated aging, linear regression was performed on the data. This linear regression found the best fit line for Td5% against aging temperature and aging time, and is displayed for the four formulations in Figure 11. Additionally, the listed slopes in the figure provide insight into how the thermal stability of the formulations will change over their lifespan. From Figure 11, all the modified formulations become more thermally stable for increasing aging temperatures while the Control remains steady. The largest changes came from the TDS-modified samples, which experienced a 10% (40 °C) increase in thermal stability. Control samples aged in air had similar thermal stability as those aged in nitrogen and the 0p2 samples aged in air had an increase in thermal stability when compared against those aged in nitrogen.

The glass transition temperature for all the formulations remained at −120 °C, which was expected, as the aging process occurs well above the glass transition. A similar linear regression treatment was performed for the coefficient of thermal expansion of the aged samples, however no clear trend existed. At the end of the 12 months of aging, all the samples fell within a range of 200–260 ppm K^−1^.

## 4. Conclusions

This work examined how small chemical changes to Sylgard 184 affected its curing behavior at room temperature. Modified formulations were created using small amounts of the Asbhy–Karstedt catalyst (a complex of 2% Pt(0) in cyclomethylvinylsiloxanes) and TDS (tetrakis (dimethylsiloxy) silane)—both of which bolster the hydrosilylation reaction that provides curing in the Sylgard framework. The Ashby–Karstedt catalyst introduces more vinyl groups and platinum while TDS introduces more silane reactive groups. It was found that all the modified formulations exhibited a dramatic reduction in working time by over 85% (less than 1.5 h) at room temperature, and that they had a greater degree of crosslinking than the Control.

Thermal accelerated aging was performed on the four formulations (Control, 0pt2, 1pt1, and 1pt2) in isothermal conditions at 25 °C, 50 °C, 60 °C, 70 °C, 80 °C, and 90 °C—spanning time periods of two, five, and twelve months. Additionally, the Control and 0pt2 samples were aged under controlled nitrogen and air atmospheres so that the evolving gases could be probed. Mass spectrometry data showed that hydrogen, methane, and ethane were the major volatile components of Sylgard 184. Two mechanisms were proposed to be responsible for aging. The hydrosilylation reaction is most responsible for the post-cure, and thus changes to solvent swelling and mechanical behavior, while trace water present throughout the polymer network explained the volatile products. The Flory–Rehner equation, along with the solvent swelling and mechanical data, demonstrate that post-curing ceases after two months of thermal aging. Additionally, using shift factors from the time–temperature superposition principle, master curves were developed for volatile evolution and Shore A Hardness, which can be used to predict lifetime material performance. Mechanical tests demonstrated that all the formulations converge to specific limiting values, where all the samples became stiffer and harder over time—with the Control exhibiting the greatest change overall. Indeed, the Control, 0pt2, 1pt1, and 1pt2 samples increased in Shore A Hardness after thermal aging by 72%, 40%, 26%, and 20%, respectively. Thermal stability tests showed that the modified samples had an increase in their onset of thermal degradation over aging times and that the Control samples did not change significantly. The largest increase in thermal stability was observed in the TDS samples, which had a 10% (40 °C) increase in thermal stability, from 350 °C to 390 °C.

Comparing the four formulations from an initial and aging standpoint, it is clear that the modified samples experience less aging. Indeed, the Control samples demonstrated a greater percent change in solvent swelling and every mechanical property examined (Shore A Hardness, maximum elongation, maximum stress, and Young’s modulus), thus illustrating that the material properties of the modified samples are more consistent throughout their lifespan. Additionally, because the material properties vary, Sylgard 184 can be altered to fit specific applications. This extends to volatile gas production, where the formulation and aging atmosphere had an effect on which gases evolved, and in varying quantities. In essence, Sylgard 184 can be tailored with small amounts of well-known compounds to optimize material properties and lifetime performance.

## Figures and Tables

**Table 1:** The formulations of Sylgard 184 used in this study.

Formulation Name	wt% TDS	wt% Asbhy—Karstedt Catalyst
Control	0	0
0pt2	0	0.2
1pt1	1	0.1
1pt2	1	0.2

**Table 2:** Final mechanical properties of the four Sylgard 184 formulations.

Formulation	Maximum Elongation (%)	Maximum Stress (MPa)	Young’s Modulus (MPa)	Shore A Hardness (ShA)
Control	74 ± 3	10.4 ± 0.7	5.74 ± 0.27	64.3 ± 0.3
0pt2	65 ± 5	8.8 ± 1.4	6.23 ± 0.32	66.4 ± 0.5
1pt1	70 ± 3	10.8 ± 1.1	6.86 ± 0.23	67.3 ± 0.8
1pt2	63 ± 3	9.3 ± 1.2	6.91 ± 0.40	68.4 ± 0.7

## Data Availability

The authors confirm that the data supporting the findings of this study are available in the article.

## Appendix A

(A1)
$$ShA\left(a_{T}t\right)=\frac{ShA^{\infty }}{1+e^{-k\left(a_{T}t-a_{T}t_{1/2}\right)}}$$

**Table A1:** Time–temperature superposition Shore A Hardness master curve model parameters.

Formulation	$\mathrm{Final}\;\mathrm{Hardness}\;(ShA^{\infty })$	Steepness (k)	$\mathrm{Midpoint}\;(a_{T}t_{1/2})$
Control	62.73	0.0013	−300
0pt2	63.98	0.0020	−525
1pt1	67.34	0.0019	−700
1pt2	68.82	0.0015	−1050

Final hardness$\;ShA^{\infty }$ values were averaged over every measurement that ended up being associated with a shift time of over 9000 h. The values presented in the table maximize R^2^.
